# The beginning of the end for chimpanzee experiments?

**DOI:** 10.1186/1747-5341-3-16

**Published:** 2008-06-02

**Authors:** Andrew Knight

**Affiliations:** 1 Animal Consultants International, 91 Vanbrugh Court, Wincott St., London, SE11 4NR, UK

## Abstract

The advanced sensory, psychological and social abilities of chimpanzees confer upon them a profound ability to suffer when born into unnatural captive environments, or captured from the wild – as many older research chimpanzees once were – and when subsequently subjected to confinement, social disruption, and involuntary participation in potentially harmful biomedical research. Justifications for such research depend primarily on the important contributions advocates claim it has made toward medical advancements. However, a recent large-scale systematic review indicates that invasive chimpanzee experiments rarely provide benefits in excess of their profound animal welfare, bioethical and financial costs. The approval of large numbers of these experiments – particularly within the US – therefore indicates a failure of the ethics committee system. By 2008, legislative or policy bans or restrictions on invasive great ape experimentation existed in seven European countries, Japan, Australia and New Zealand. In continuing to conduct such experiments on chimpanzees and other great apes, the US was almost completely isolated internationally. In 2007, however, the US National Institutes of Health National Center for Research Resources implemented a permanent funding moratorium on chimpanzee breeding, which is expected to result in a major decline in laboratory chimpanzee numbers over the next 30 years, as most are retired or die. Additionally, in 2008, *The Great Ape Protection Act *was introduced to Congress. The bill proposed to end invasive research and testing on an estimated 1,200 chimpanzees confined within US laboratories, and, for approximately 600 federally-owned, to ensure their permanent retirement to sanctuaries. These events have created an unprecedented opportunity for US legislators, researchers, and others, to consider a global ban on invasive chimpanzee research. Such a ban would not only uphold the best interests of chimpanzees, and other research fields presently deprived of funding, but would also increase the compliance of US animal researchers with internationally-accepted animal welfare and bioethical standards. It could even result in the first global moratorium on invasive research, for any non-human species, unless conducted in the best interests of the individual or species.

## Introduction

### Ending US chimpanzee experimentation

On 17^th ^April, 2008, a bi-partisan political group introduced *The Great Ape Protection Act *to US Congress. The bill proposed to end invasive research and testing on an estimated 1,200 chimpanzees confined within US laboratories – some for over 40 years. For approximately 600 federally-owned, the bill would also ensure permanent retirement to sanctuaries [1].

Congressman Roscoe Bartlett, who – along with others – introduced this new bill, stated: *"As a scientist who worked with chimpanzees on research projects, I believe the time has come to limit invasive research on these animals and rigorously apply existing alternatives."*

Within the US, laboratory chimpanzee numbers had previously soared when the National Institutes of Health (NIH) implemented a breeding program in 1986 to meet the demands of researchers seeking to study the newly-emergent acquired immunodeficiency syndrome (AIDS) epidemic. Following the failure of the chimpanzee model to produce clinically useful outcomes, however, in 2007 the NIH National Center for Research Resources (NCRR) made permanent a breeding moratorium temporarily implemented in 1995 [2,3]. Numbers steadily declined thereafter, and by October 2006, 1,133 chimpanzees remained within six US primate centers [2].

Finances played a large role in the NCRR decision to reduce chimpanzee numbers. With captive chimpanzees living an average of 30 (males) to 45 (females) years, the lifetime costs of supporting them are estimated as between $300,000 and $500,000 [3]. NCRR figures indicate that 650 federally-funded chimpanzees will cost a total of $325 million to support [2]. Although privately-funded research chimpanzees remain unaffected, the NCRR decision is nevertheless expected to result in a major decline in laboratory chimpanzee numbers over the next 30 years, as most are retired or die.

Hence, whether through legislation or budgetary restriction, invasive chimpanzee research may be drawing to a close within the US. Furthermore, these events may herald the beginning of the end for chimpanzee experimentation internationally. Although around half a dozen other countries also conducted chimpanzee experiments 15 years ago, by 2008 the US stood virtually alone. Every other country except perhaps Gabon – whose future plans were unclear – had ceased invasive chimpanzee experimentation. Should the US and Gabon also terminate such research, it would effectively result in the first global moratorium on invasive research for any non-human species, unless conducted in the best interests of the individual or species.

### International bans on great ape experimentation

The NCRR decision followed a campaign by the Humane Society of the US which resulted in nearly 22,000 letters to the NCRR [4]. Other US animal protection organizations such as the New England Anti-Vivisection Society [3] and In Defense of Animals [5], and international organizations such as the British Union for the Abolition of Vivisection [6], have similarly initiated campaigns against chimpanzee or great ape (chimpanzees, bonobos, gorillas and orang-utans) experimentation, in recent times. This increasing focus of animal protection organizations is mirrored by increasing public concern about invasive experimentation on chimpanzees and other great apes, given their relatively advanced sensory, psychological and social capabilities. This concern has contributed to a growing number of national bans against invasive experiments on great apes.

In the UK, special justifications for experiments on great apes became necessary under the *Animals (Scientific Procedures) Act 1986*, and in 1997 a policy ban was placed on such experiments by the Home Office [7-9]. Great ape experimentation has also been banned in Sweden (regulatory restrictions since 2003, with the exception of non-invasive behavioral studies), and Austria (since 2006, unless conducted in the interests of the individual animal). Unusually, the Austrian ban also protects gibbons, which is in line with current taxonomic classification including gibbons within the family Hominidae [3,9]. The Netherlands was the last European country to conduct invasive research on chimpanzees. It outlawed great ape experimentation from 2004 [3,9].

In countries such as Italy and Norway, great apes have not been used for years, although national bans have yet to be passed. Since 1992 (personal communication: Kolar R, German Animal Welfare Academy, Neubiberg; 17 Apr. 2008), great apes have not been subjected to invasive research within Germany, although non-invasive cognitive and behavioral studies do occur. In 2002, the Belgian minister responsible for animal welfare announced that Belgium would be working toward a ban on all primate experiments, and a Swiss state ethics commission recently demanded that the Swiss government ban great ape experimentation [3,6,9-14].

Japan ceased invasive research on chimpanzees in 2006 [15]. In Australia and New Zealand, great ape experimentation is restricted by policy (Australia) [16], or legislation (New Zealand, since 1999) [9,17]; unless in the best interests of the individual animal or species.

Related developments continue to occur internationally. The European Principality of Liechtenstein imposed a total ban on animal experiments in 1989 [18]. In 2007, the Republic of San Marino similarly banned all animal experiments [19-21], and the Balearic Islands – one of the Autonomous Communities of Spain – granted basic legal rights to great apes. Due to the popularity of this development, the Spanish government was considering expanding it to include all of Spain [22].

In late 2007, 433 Members of the Members of the European Parliament (MEPs) signed *Parliamentary Written Declaration 40/2007*, calling for urgent action to end the use of great apes and wild-caught monkeys in experiments, and for the establishment of a timetable for the cessation of all European primate experiments. This number of signatories was the highest recorded for any Written Declaration on an animal protection issue, and the third highest for a Declaration of any kind, since 2000. The Declaration must be formally considered by European Commission officials when drawing up applicable legislation. It calls for changes to *European Directive 86/609/EEC on the Protection of Animals used for Experimental and Other Scientific Purposes*, which governs animal use within European Union (EU) member states. By mid 2008, a formal revision of *Directive 86/609/EEC *was nearing completion. The Directive presently allows experiments on all non-human primates (NHPs), around 10,000 of which are subjected to experiments within Europe annually, with the greatest recorded use occurring within the UK, followed by France and Germany [23,24].

Stated UK MEP Dr Caroline Lucas, *"The EU is currently reviewing its rules on laboratory animals, and we must use this opportunity to immediately ban the use of primates in experiments anywhere in the EU, in favour of more modern and effective alternatives like computer modelling, tissue or cell cultures and micro-dosing" *[24].

Swedish MEP Jens Holm similarly stated, *"It is time to end experiments on primates. Primates are sentient beings and are fully capable of having feelings like humans: joy, happiness or anger. Their interests must be fully taken into account, and cruelty against them must stop." *[24].

### Chimpanzee 'personhood'? A legal challenge

In Austria a noteworthy campaign to grant legal protection to an individual chimpanzee was being hotly contested in 2007 – 2008. Based on scientific argument that chimpanzees possess a 'theory of mind,' and ought to be classified within the genus *Homo*, advocates sought recognition under Austrian law of the 'personhood' of a chimpanzee named Matthew Pan.

Matthew has passed a mirror self-recognition test, demonstrated tool use and understanding, drawn pictures, and played with human caretakers. His advocates assert that chimpanzees in general, and Matthew in particular, have demonstrated ample evidence consistent with a theory of mind [25-29].

However, theories of mind are scientifically controversial for animals [30]. Recent evidence that suggests that in many respects chimpanzees possess the necessary mental characteristics, whereas in others they might not. Solid evidence exists that chimpanzees understand the goals, intentions, perception and knowledge of others. Nevertheless, despite several apparently valid attempts, evidence remains lacking that they understand false beliefs. It therefore appears chimpanzees may understand others in terms of a perception-goal psychology, as opposed to the belief-desire psychology more characteristic of humans [31]. Ongoing scientific interest within this field is evidenced by recent symposia [32].

It nevertheless remains clear that chimpanzees in general, and Matthew in particular, possess the ability to reason, and to recognize the interests of others, which are key requirements, Matthew's advocates argue, for recognition of 'personhood' under Austrian law.

The proposed re-classification of chimpanzees within the genus *Homo *rests on the very high degree of similarity between chimpanzee and human DNA [33,34]. Advocates state that the similarities are roughly equivalent to those of some other species classified within the same genus, such as certain equines [29]. However, critics assert that the remaining differences result in neuroanatomical, other morphological, cognitive, behavioral and additional phenotypic variation sufficient to justify the unique taxonomic classification of humans [35,36].

If Matthew's advocates ultimately succeed on his behalf, Matthew could no longer be legally considered property. He would also become eligible for legal guardianship, on the basis that he was abducted as an infant, involuntarily confined in an alien environment for most of his lifetime, and is consequently unable to fend for himself, or safeguard his own interests. Through such a guardian Matthew would be able to receive donations toward his living costs, and even – potentially – sue those responsible for his capture in West Africa in 1982, for AIDS and hepatitis research [29,37,38]. Matthew's case is highly controversial, and the legal and philosophical ramifications would be enormous, should his case be upheld.

By early 2008, Matthew's legal advocates had not succeeded within the Austrian courts, and were planning to pursue his case within the European Court of Human Rights. Whether or not they are ultimately successful, rapidly growing interest in this field – including the publication of detailed legal foundations supporting the legal 'personhood' of chimpanzees [39] – strongly suggests that Matthew's case is unlikely to be the last of its kind.

### Retirement of laboratory chimpanzees

Opinion is also growing that chimpanzees should be retired at the end of their involvement in biomedical research, into sanctuaries capable of providing for their social and psychological well-being, for the remainder of their natural lives [5,40-43]. The US *Chimpanzee Health Improvement, Maintenance and Protection Act 2000*, for example, requires that chimpanzees no longer needed for biomedical research be retired to sanctuaries. The US *Chimp Haven is Home Act 2007 *repealed provisions of the *Public Health Service Act 1946 *that had permitted the removal of chimpanzees from the federal sanctuary system, for research purposes. The *Chimp Haven is Home Act *prohibits the use of such chimpanzees for research, other than within noninvasive behavioral studies [44].

Perhaps the first large-scale retirement of laboratory chimpanzees occurred in 2002, when Baxter Healthcare Corporation transferred over 40 chimpanzees, and more than 80 monkeys, from its Hans Popper Primate Center (HPPC) outside of Vienna, Austria, to the Home of Primates – Europe, Safaripark, Gänserndorf, Austria. For many years the Center had used these primates to test putative vaccines for viruses such as hepatitis B and C (HBV and HCV), HIV, and therapeutic plasma proteins such as Factor VIII. Both the advent of alternative testing systems and a change in research focus led to Baxter's 1998 decision to end non-human primate testing at HPPC and seek a permanent retirement site [45].

In 2002 the Netherlands agreed to fund the re-homing of chimpanzees by the charitable foundation Stichting Aap [42]. The Dutch national colony of over 100 chimpanzees were relocated to sanctuaries, zoos and safari parks. In 2007 similar efforts were underway in Japan [3].

Within the US, the New York Blood Centre (NYBC) plans to retire 74 chimpanzees used in its hepatitis research programs at its Vilab laboratories in Robertsfield, Liberia. The chimpanzees will be retired to six remote African islands, purchased from the Liberian Government to provide a sanctuary [46]. The NYBC no longer considers such chimpanzee experiments acceptable on ethical and welfare grounds, believing that *"there are new methods for doing this kind of research." *[3,47].

### Calls for increased chimpanzee use

Apparently seeking to counter increasing international opinion against invasive chimpanzee experimentation, advocates have recently begun extolling its alleged benefits, calling for its continuation. In a recent, prominent plea in *Nature *for increased funding for such research, several heads of US primate research centers stated that chimpanzee experimentation has been of critical importance during struggles against major human diseases [48]. Similarly, British scientists recently called for the right to conduct such research on chimpanzees, contrary to the existing UK ban, in rare scenarios, such as the investigation of dangerous emerging infectious diseases [49].

## Discussion

Rapid international developments within this field justify a re-examination of the merits of invasive chimpanzee experimentation. Such a reappraisal is most applicable to the US. Although US animal research is governed by international, federal, and state laws, regulations, rules, guidelines, and standards [50], contrary to the legislation of other key countries, the US *Animal Welfare Act 1966 *(most recently amended in 1990) does not require the use of non-animal alternatives, even when scientifically validated alternatives exist. Unsurprisingly, therefore, US primate use is more than five times in excess of the number used in the entire European Union (approximately 58,000 vs. 11,000 annually; [13]).

### Advancements in biomedical knowledge?

When assessing the merits of invasive chimpanzee experimentation, a necessary first step is to obtain a definitive overview of the disciplines investigated by such research. Accordingly, I recently surveyed three major biomedical bibliographic databases and examined published studies conducted worldwide from 1995 – 2004 [51].

I sought to assess the value of research on captive chimpanzees, particularly when invasive – that is, involving the entry of a needle, catheter or other instrument within the body, by puncture or incision – because such research incurs the greatest bioethical and social concerns. I included studies of captive chimpanzees or their tissues, and excluded studies of free-living populations, veterinary medical case reports of naturally-ill chimpanzees – whether or not in captivity, most genome studies, studies of skeletal anatomy – which frequently used museum specimens, and studies of cell lines (although I did include cell samples, such as peripheral blood mononuclear cells, obtained from captive chimpanzees).

**Figure 1:**
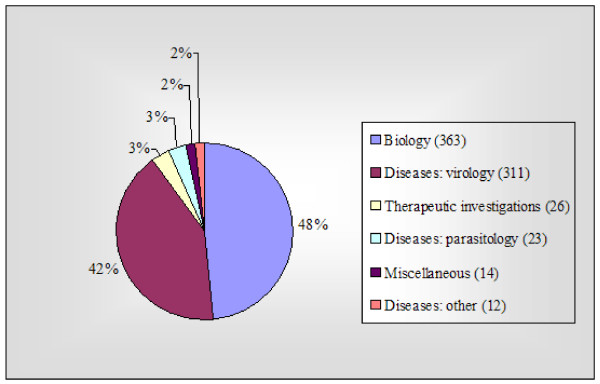
Chimpanzee experiments 1995–2004 (total 749).

749 studies of chimpanzees or their tissues were located that met my inclusion criteria, of which 48.5% (363/749) were biological experiments, and 41.5% (311/749) were virological experiments (Figure 1).

**Figure 2:**
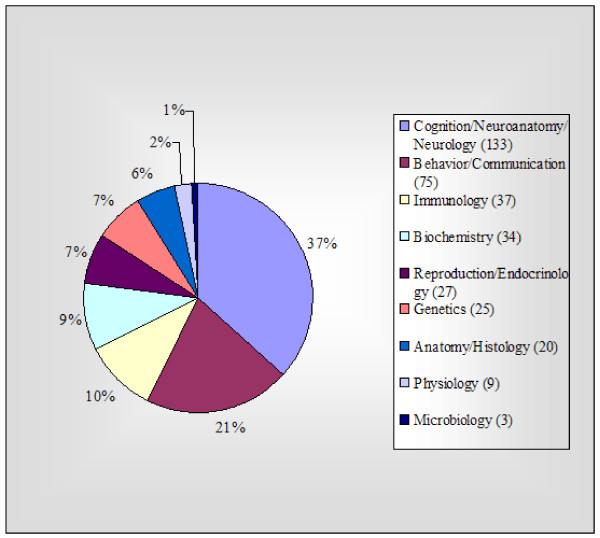
Biology experiments (363 of 749).

Biological studies were conducted within nine broad disciplines (Figure 2), of which the most common were cognition/neuroanatomy/neurology (36.6%, 133/363), and behavior/communication (20.7%, 75/363).

**Figure 3:**
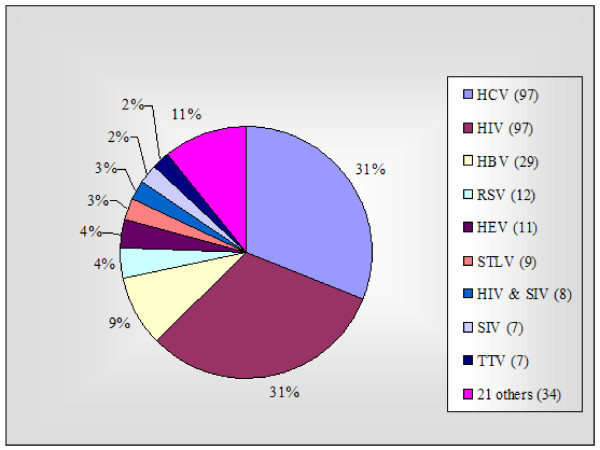
**Virology experiments (311 of 749)**. 21 others: Six: FV. Four: HAV. Two each: GBV – B, HIV & HV, IV, PIV, Noroviuses. One each: Bacteriophages, Dengue v., Ebola v., HCMV, HGV, HMPV, H/S TLV, LCV, Papillomaviruses, RV2, Rhinovirus, VZV, WMHBV, Unspecified. HCV = hepatitis C v., HIV = human immunodeficiency v., HBV = hepatitis B v., RSV = respiratory syncytial v., HEV = hepatitis E v., STLV = simian T-cell lymphotropic v., SIV = simian immunodeficiency v., TTV = transfusion-transmitted v., FV = foamy v (human and simian FV), HAV = hepatitis A v., GBV-B = GB virus B, HV = herpes v., IV = influenza v., PIV = parainfluenza v., HCMV = human cytomegalovirus, HGV = hepatitis G v., HMPV = human metapneumovirus, H/S TLV = human/simian T-cell leukemia v., LCV = lymphocryptoviruses, RV2 = rhadinovirus (or gamma-2-herpesvirus) genogroup 2, VZV = varicella-zoster v., WMHBV = woolly monkey hepatitis B v.

41.5% (311/749) of all chimpanzee experiments were virological studies. Thirty viruses were investigated, of which the most frequent were HCV and HIV (both 31.2%, 97/311) (Figure 3).

The remaining experiments comprised therapeutic investigations (3.5%, 26/749) – namely, pharmacological, toxicological and anesthesiological investigations, and the testing of surgical techniques or prostheses; investigations of eight parasitic species (3.1%, 23/749) – of which the most frequent were the malaria protozoa *Plasmodium falciparum *and *P. ovale *(26.1%, 6/23), the roundworm *Onchocerca volvulus *(21.7%, 5/23), and the flatworm *Schistosoma mansoni *(17.4%, 4/23); and other diseases and miscellaneous experiments, which jointly comprised 3.5% (26/749) of all chimpanzee experiments.

**Figure 4:**
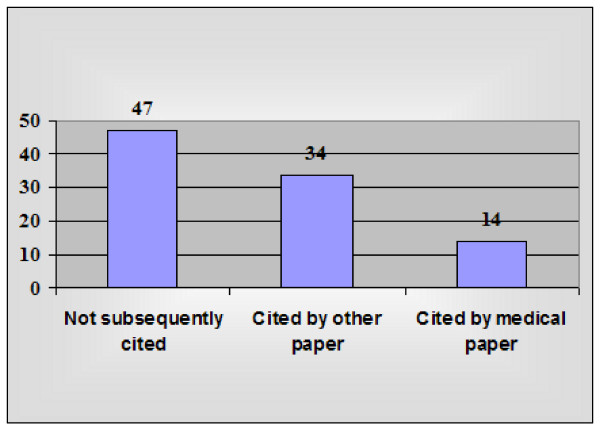
Citations of 95 randomly-selected published chimpanzee studies.

On the face of it, these studies appear to have contributed toward a large array of biomedical disciplines. However, not all knowledge has significant value, nor is worth the bioethical, financial or other costs that may be incurred in gaining that knowledge. To gain a more critical assessment of the utility of invasive chimpanzee research in advancing biomedical knowledge, I randomly selected a statistically-significant subset of 95 experiments, and determined the frequency with which they were cited by papers subsequently published and included within these comprehensive bibliographic databases. 49.5% (47/95; 95% CI = 39.6 – 59.4%) were not cited by any subsequent papers (Figure 4).

Given that almost all of these chimpanzee experiments would have been approved by at least one institutional ethics committee (Institutional Animal Care and Use Committee – IACUC – within the US), entrusted with ensuring that their welfare-related, bioethical and financial costs were reasonably likely to be exceeded by their expected benefits, it is disturbing that half of these randomly-selected experiments were not cited by any subsequent papers. The year of publication did not appear to substantially affect this outcome, as citation frequencies were similar across the decade, with more recent papers cited approximately as often as older papers.

Citation frequencies are not, of course, a definitive indication of the benefits or lack thereof, of scientific research. Uncited studies may also contribute to the advancement of biomedical knowledge, through a variety of mechanisms. However, citation frequencies do generally provide a quantifiable and reasonably objective approximation of utility, or lack thereof. Research that makes a significant contribution to a field – such as by confirming or refuting hypotheses – is very likely to be cited by future papers, as is research that produces interesting or controversial outcomes. On the other hand, research that is inconclusive, or of little interest or significance, is much less likely to be cited.

The disappointing citation rate of these chimpanzee studies is therefore cause for considerable concern. It is unreasonable to conclude that a large number of studies made a significant contribution, if none were cited by any future publication, as occurred for half of these randomly-selected chimpanzee studies.

Given that much research of lesser significance is not published, these published chimpanzee experiments can generally be assumed to be those with the greatest potential for advancing biomedical knowledge. Consequently, these results indicate that the majority of invasive chimpanzee studies generate data of questionable value, which makes little obvious contribution toward the advancement of biomedical knowledge.

### Advancements in human healthcare?

Most would consider that the greatest justifications for invasive chimpanzee research involve attempts to advance human health. As stated, advocates of such research claim it has been of critical importance during our struggles against major human diseases [48]. To critically assess such claims, I determined the frequency with which the statistically-significant subset of 95 randomly-selected chimpanzee studies had been cited by papers describing prophylactic, diagnostic or therapeutic methods efficacious in combating human diseases.

Only 14.7% (14/95; 95% CI = 8.9 – 23.4%) of all invasive chimpanzee studies were cited by a total of 27 papers describing well-developed diagnostic methods (5) or prophylactic and/or therapeutic methods (22) for combating human diseases (Figure 4). Diseases examined included cancer (non-specific), chronic obstructive pulmonary disease, Epstein-Barr virus, hepatitis viruses A through G, hepatocellular carcinoma, HIV, malaria, organ transplant rejection, respiratory syncytial virus, rheumatoid arthritis, rhinovirus colds, systemic lupus erythematous, and transmissible spongiform encephalopathies.

As stated, citation rates are not a definitive indication of utility or lack thereof. Invasive chimpanzee studies may have contributed to medical advances through various indirect means, such as by stimulating investigation of certain lines of inquiry in humans – although it is unlikely that any medical papers subsequently published would not cite the chimpanzee studies that provided such inspiration. Alternatively, chimpanzee studies may have contributed to investigations of disease etiology, or to papers describing prophylactic, diagnostic or therapeutic methods for combating human diseases in early stages of development – although potential human benefits, in such cases, remain speculative.

However, it is reasonable to expect that if chimpanzee research had truly been of critical importance during struggles against major human diseases, as claimed by advocates, such chimpanzee studies would, in fact, be cited by papers describing methods efficacious in combating those diseases. The only alternative is that none of the struggles to which chimpanzee research purportedly made major contributions, resulted in effective, published solutions.

In fact, 27 papers describing well developed prophylactic, diagnostic or therapeutic methods for combating human diseases *did *cite chimpanzee studies. However, detailed examination of these medical papers revealed that *in vitro *studies, human clinical and epidemiological studies, molecular assays and methods, and genomic studies, contributed most to their development.

The randomly-selected chimpanzee studies proved to be of peripheral importance to most of these medical papers, for a variety of reasons. 63.0% (17/27) were, in fact, wide-ranging reviews of 26–300 (median 104) references, to which the cited chimpanzee study made a very small contribution.

In 12 cases the chimpanzee studies appeared redundant, as humans or human sera were studied concurrently, or because they served only to confirm previous human observations. In seven cases the method explored in the cited chimpanzee study was not developed further, sometimes because later clinical trials in humans failed to demonstrate safety or efficacy, contrary to positive chimpanzee results. In five cases the chimpanzee study examined a disease or method of only peripheral relevance to the medical method described. In three cases the chimpanzee study merely illustrated an historical finding, or was cited only during historical discussions of attempts to combat the disease in question. In another three cases the chimpanzee studies yielded results inconsistent with data from other NHP studies, and in two cases they yielded results inconsistent with human data. In two more cases only the human outcomes from human studies concurrently described within the cited chimpanzee studies were discussed. In one case the chimpanzee study may have helped establish the need for a new diagnostic method, but did not contribute further to its development.

In fact, none of these cited chimpanzee studies demonstrated an essential contribution, or – in most cases – a significant contribution of any kind, toward the development of the medical method described.

### Limitations on the medical utility of chimpanzee models

These results suggest a lack of utility of chimpanzees as experimental models for studying human diseases. On the face of it, this appears counter-intuitive, given the genetic similarities of chimpanzees and humans. Our two species shared a common ancestor just 5–7 million years ago [52] – a very short period in phylogenetic terms.

A 2005 draft of the chimpanzee genome confirmed it to be 98.77% identical to the mean human genome in terms of base pairs [53]. When considering only the most functional DNA – that is, bases that cannot be altered without a consequent change in the amino acid coded for by the gene, as distinct from bases that may be altered without such changes, or so-called 'junk' DNA outside coding regions – Wildman and colleagues [33] found a 99.4% correlation between chimpanzees and humans. However insertions, deletions and consequent misalignments raise the total estimated difference to around 4–5% [54,55].

While a minority of these genetic differences lie within structural genes, most are now known to lie within the regulatory regions of our DNA. By controlling the activities of structural genes, regulatory genes can exert an 'avalanche' effect upon hundreds of other genes. Consequently, a small difference may have profound effects [56]. Striking differences have been found in the levels of gene expression between chimpanzees and humans, within the brain and liver, for example [57]. Although chimpanzees and humans differ in only 4–5% of their DNA, that difference is sufficiently important to result in a difference of around 80% in protein expression [58], yielding marked phenotypic differences between the species.

Additionally, systemic responses to disease agents and test chemotherapeutics within laboratory chimpanzees may be distorted by the neurological, endocrinological and immunological abnormalities that may result from a variety of experienced stressors. Although when between studies chimpanzees may be housed within social groups, with access to enlarged, environmentally-enriched enclosures, during study participation laboratory housing may be small, barren and standardized, and chimpanzees may experience isolation, trauma, chronic boredom, and a variety of stressful laboratory procedures [59].

The substantial differences in protein expression between chimpanzees and humans, and the further distortions of normal physiology that may result from stressful laboratory environments and procedures, confer differences in the susceptibility to, etiology and progression of various diseases; differing absorption, tissue distribution, metabolism and excretion of chemotherapeutic agents; and differences in the toxicity and efficacy of pharmaceuticals.

Whilst it is true that interspecies differences in response may sometimes be illuminating – for example, during elucidation of disease mechanisms – it is generally more desirable that experimental models mimic human responses as accurately as possible, within experiments aimed toward the development of prophylactic, diagnostic, or therapeutic methods for combating human diseases. The lack of *fidelity*, or accurate reproduction, of key human characteristics and responses, is the most likely cause of the demonstrable lack of utility of chimpanzee models during the development of methods efficacious in combating human diseases.

### Bioethically-relevant chimpanzee characteristics

Chimpanzees possess a range of advanced sensory, psychological and social characteristics, which may enhance their potential for suffering, and are therefore morally relevant when considering the ethics of subjecting them to invasive experimentation.

### Pain perception

Whilst chimpanzees lack some of the most advanced human neurological and cognitive capacities [60], it nevertheless remains true that they possess well-developed neuroanatomical mechanisms common to vertebrates – including nerve endings ('nociceptors') and peripheral and central neuroanatomical architecture – that confer the ability to detect and perceive as painful a variety of noxious stimuli, including mechanical, chemical and thermal insults. Such mechanisms evolved partly to encourage avoidance of natural agents capable of causing tissue damage. These same mechanisms may result in pain perception when chimpanzees are exposed to invasive procedures, noxious stimuli, or tissue damage secondary to artificially-inflicted diseases or toxic agents.

Most – if not all – invasive experiments result in at least mild physical discomfort, for example, during restraint and venipuncture, and some may result in marked discomfort or pain. Whilst analgesic provision is adequate in some cases, it is less so in others, partly due to concerns – well-founded or otherwise – that experimental outcomes may be altered by drug use. Whilst anaesthetic and analgesic use undoubtedly alters normal physiology, claims that such alterations are sufficiently important to hypotheses under investigation, to warrant their exclusion, require careful scrutiny. Despite increasing recognition [61,62] that pain relief improves both animal welfare and research quality – via minimization of pain-related physiological, psychological, behavioural or other animal model distortion – pain monitoring and analgesic provision remains less than optimal within many animal research protocols [63,64].

### Emotional capacity

The potential suffering of laboratory chimpanzees is compounded by their relatively advanced emotional capabilities. They appear able to experience a range of emotions, similar to those we label as happiness and sadness, fear and anxiety, irritation, rage and despair [65-67], and appear able to suffer emotional, as well as physical, pain [59]. Psychological stress is likely to result both from aversive experiences directly, and from the inability of laboratory chimpanzees to escape them. Such considerations are of greatest concern where pain or discomfort are substantial or prolonged.

### Psychological abilities

The relatively advanced capacities of chimpanzees to understand and remember that certain people, tools or procedures are likely to cause pain and distress, and their ability to anticipate future aversive experiences, is likely to compound the distress such events may cause. Chimpanzees have some capacity to anticipate and understand the intentions and psychological states of others [68-71], and have long memories [67,72-75]. The psychological abilities of chimpanzees may encompass abstract reasoning [76], self-awareness (although mirror self-recognition may decline with age) [66,77,78], and simple problem solving [67,79]. These relatively advanced abilities most probably evolved to enable chimpanzees to cope with their complex natural environments and social structures [66].

### Social characteristics

Chimpanzees are highly social animals, and the disruption of social networks when animals are captured from the wild – as many older research chimpanzees once were – or when subjected to confinement or translocation during biomedical research, may add to their suffering. The social relationships of chimpanzees appear to encompass prolonged rearing of offspring, close and affectionate family bonds, friendship, and mourning behavior following the deaths of companions [66]. Anecdotal accounts of consolation of victims of aggression, and solicitous treatment of injured individuals, suggest that chimpanzees feel empathy [80,81]. Chimpanzees plan for the future and interact in a variety of cooperative activities, including territorial patrols, coalitionary aggression, cooperative hunting, food sharing and joint mate guarding [72,73,81].

Chimpanzees possess well-developed communicative skills. Facial expressions [82] and sophisticated vocalizations [83-85] convey information, for example, about identity [83], emotional states [82] and social status [84]. Chimpanzees kiss, hold hands, pat one another on the back, embrace, tickle, punch and swagger [66], with gestural dialects varying between communities [86].

Although chimpanzees appear to lack the ability to explicitly teach [60], they have some ability to learn through observation, emulation and practice [87], although limitations on learning capacity have been recorded [88]. At least 39 behavior patterns, including courtship, grooming, tool manufacturing and use, essentially comprising discreet 'cultures,' are passed from generation to generation through such learning. As with human cultures and customs, these have been shown to vary substantially between chimpanzee communities [8,89], in ways that cannot be attributed solely to ecological or genetic variation [90].

De Waal [91,92] asserted that the social sophistication of chimpanzees is similar to that of humans, and that reciprocity among them is influenced by a similar sense of moral 'rightness' and justice. Chimpanzees may reject exchanges in which they value potential gains less than potential losses, for example, but, as with humans, there is no evidence that they are averse to interactions from which they benefit [81].

Despite the relatively advanced sensory, psychological and social sophistication of chimpanzees, certain morally-relevant dissimilarities with humans do exist. Recent research suggests that human altruistic behavior – that is, a willingness to incur costs to assist genetically-unrelated strangers, in the absence of any personal gain ('other-regarding preferences') – provides a key example. In contrast, assistance offered by chimpanzees and other NHPs appears mainly limited to biologically-related or reciprocating individuals, and is rarely extended to unfamiliar individuals [81,93], although such behavior has been observed in common marmoset monkeys (*Callithrix jacchus*) [94].

### Bioethical considerations

During study participation chimpanzees may be individually confined within small, relatively barren, standardized cages [67], on the assumption that these facilitate cage cleaning, minimize infection risks, and facilitate ease of access, such as for blood sampling. They may be housed in buildings lacking windows, without access to natural lighting [66]. They may be involuntarily subjected to potentially harmful experiments, including the artificial induction of diseases, and tests of the toxicity and efficacy of chemotherapeutic agents.

It is reasonable to expect that the relatively advanced sensory, psychological and social characteristics of chimpanzees may enhance their capacity for suffering during involuntary participation within invasive research protocols – and particularly, overtly harmful research. In the opinion of some experts, such chimpanzee characteristics render it impossible to provide laboratory environments that satisfactorily meet their minimum physiological and behavioral requirements [8,95].

Unanswered questions about the precise psychological abilities of chimpanzees inevitably result in a degree of uncertainty about the nature and magnitude of the suffering likely to result from such protocols and procedures. However, where such doubt exists, it seems reasonable to apply a precautionary principle – assuming that suffering *may *occur, and considering restrictions on procedures likely to cause such suffering – until proven otherwise. Such precautionary principles are, after all, enshrined within other fundamental social institutions, because they are considered to be rational, reasonable and humane. The Western legal system, for example, generally assumes innocence until guilt is proven beyond reasonable doubt. Where such doubt remains, judicial punishment is withheld.

Conversely, however, a precautionary principle might also be applied in favor of human patients or consumers that may potentially benefit from laboratory animal experimentation: where healthcare advances or other human utility *may *result, perhaps such experiments should proceed, until lack of potential benefit is proven beyond reasonable doubt.

When applied in isolation, each of these viewpoints represents a diametrically opposite position, consistent with ideological viewpoints that consider the interests of animals, or people, respectively, as overwhelmingly more important than those of each other. Such viewpoints are but two of a diverse range of religious, cultural and philosophical viewpoints about our moral duties toward animals and people that could be applied [96-98].

It is the opinion of this author, however, along with various philosophers [98,99], that achieving a reasonable and rational balance between the interests of people and those of laboratory animals requires balanced consideration of the interests of both groups: primarily, the likely benefits accruing to humans, and the probable costs incurred by animal experimental subjects. Such a 'utilitarian' position aims to achieve the 'greatest good for the greatest number,' and considers the interests of all affected, whether human, or other creatures likely to be capable of experiencing states as 'good,' or less desirable.

Fortunately, in the case of invasive chimpanzee experimentation, it is possible to achieve a reasonable weighting of interests, because concrete evidence about the likely human benefits, and costs to chimpanzees, does exist. Invasive chimpanzee experimentation allows investigation of a virtually limitless number of scientific questions. However, as previously demonstrated, the majority of such experiments appear to generate data of questionable value, which makes little obvious contribution toward the advancement of biomedical knowledge. Additionally, such studies rarely – if ever – make significant contributions toward the development of methods efficacious in combating human diseases [51]. The resource and financial burdens incurred by such research are also considerable – issues of no small importance, within a climate of ever-increasing competition for scarce research resources.

The costs to chimpanzees enrolled in such experiments include involuntary confinement within laboratory settings, social disruption, and participation within potentially-harmful research protocols. Recent studies have established beyond any reasonable doubt that the effects of laboratory confinement and procedures, especially long-term, can be severe. Many captive great apes show gross behavioral abnormalities, such as stereotypies, self-mutilation or other self-injurious behavior, inappropriate aggression, fear or withdrawal [100,101], including among chimpanzees recently retired from US laboratories [102]. It is increasingly acknowledged that such abnormal behaviors resemble symptoms associated with human psychiatric disorders, such as depression, anxiety disorders, eating disorders, and post-traumatic stress disorder, and that pharmacological treatment modalities similar to those applied to human patients may be appropriate, and indeed, morally compelled, for severely disturbed animal patients [100,103]. Long-term therapeutic combination with positive reinforcement training, environmental enrichment, and social and environmental modification may be necessary in severe cases [101].

The analogous legal scenario is once again illuminating. Although these highly sentient creatures are in no way responsible for any human grievance, such as the serious diseases we attempt to induce in them, we sometimes subject chimpanzees to conditions that would cause widespread social outrage if used to punish the most heinous of human criminals – for years on end, and in some cases, for decades. Bradshaw and colleagues [102] observed that: *"The costs of laboratory-caused trauma are immeasurable in their life-long psychological impact on, and consequent suffering of, chimpanzees." *As stated, humans are not usually punished until proven guilty beyond reasonable doubt. It is not altogether unreasonable to assert that the lack of humanity highlighted by this difference in standards applies less to chimpanzees, than to ourselves.

The logic of Bradshaw and colleagues' corollary is elementary, yet compelling: *"In human traumatology, the first step in treatment is to arrest its causes. This implies that prevention and treatment of chimpanzee psychopathology entails considering the factors and institutions that have brought chimpanzees to the point of irreversible distress: in simple terms, desisting from using apes as biomedical subjects in lieu of humans is compelled if trauma is not to be perpetuated."*

The unique biological characteristics of chimpanzees – which are rare in their own right – and their advanced sensory, psychological and social characteristics – which have some similarities with those of humans – all create a strong ethical basis for acknowledging the necessity of respecting at least the most basic and essential interests of chimpanzees, such as their interests in avoiding death, pain, suffering and captivity [104,105]. When according due consideration to the interests of both humans and chimpanzees, it cannot be concluded that invasive chimpanzee experimentation is generally ethically justifiable.

### Acceptable chimpanzee research?

Potential chimpanzee research protocols range from field studies of free-living (wild) populations, through non-invasive behavioral or psychological studies of sanctuary or laboratory populations, to mildly-harmful invasive experimentation, more-harmful experimentation, and finally, to include research protocols resulting in major harm or death. According due respect to chimpanzee characteristics and associated bioethical considerations does not require the termination of all chimpanzee research. Bioethical concerns are minimized within non-invasive observational, behavioral or psychological studies of free-living or sanctuary populations.

It is precisely the advanced psychological abilities of chimpanzees that may incur marked welfare-related and bioethical burdens during biomedical experimentation, that also place chimpanzees at risk of boredom and associated pathology within sanctuary settings, unless highly enriched. Offering such chimpanzees the choice to participate within behavioral or psychological studies, may, in fact, constitute a valuable form of environmental enrichment [106]. Whilst participation remains truly voluntary, rather than coerced through conditional provision of essential needs, such as sufficient food, water, or social contact with compatible conspecifics, bioethical concerns are minimized. As stated, such studies are consistent with existing bans on great ape experimentation in countries such as Sweden, and consistent with the US *Chimp Haven is Home Act 2007*, which prohibits further research on chimpanzees retired to federal sanctuaries, other than non-invasive behavioral studies [44].

Limiting chimpanzee experimentation to non-invasive observational, behavioral or psychological studies of free-living or sanctuary populations would inevitably restrict the range of scientific questions that might be investigated. It would, however, strike the correct ethical balance between satisfying the interests of chimpanzees, and those of human beings.

### Ecological considerations

Alarming declines in wild chimpanzee populations [107] have led some to call for the maintenance of experimental chimpanzee populations for conservation reasons. However, chimpanzees maintained in captive environments other than very expansive, naturalistic settings, are unlikely to retain the full range of abilities, characteristics and behaviors demonstrated by wild chimpanzees. It is not only the physical characteristics of chimpanzees that are of scientific interest, or worthy of preservation, after all. For reasons such as these, the ultimate objective of genuine conservation programs is the maintenance or re-establishment of wild populations. Unfortunately, the capacity of chimpanzees to recover from disturbance is limited, and the reinforcement of wild populations with captive-born individuals is rarely a realistic option [108]. Conservation efforts are therefore most appropriately directed toward addressing the factors responsible for declining wild chimpanzee populations – particularly, habit destruction, hunting and the spread of Ebola haemorrhagic fever – through aggressive investments in law enforcement, protected area management and Ebola prevention [107].

## Conclusion

Few research issues generate as much controversy as invasive chimpanzee experimentation. The unequalled phylogenetic proximity of chimpanzees to humans makes them potentially superior to all other laboratory species for use as human models within toxicity experiments and pathological or therapeutic investigations it would be hazardous to conduct on humans. However, chimpanzees are also associated with perhaps the greatest animal welfare and bioethical concerns of any laboratory species, due to their advanced sensory, psychological and social characteristics. These confer a marked ability to suffer when born into unnatural captive environments, or captured from the wild – as many older research chimpanzees once were – and when subsequently subjected to confinement, social disruption, and involuntary participation in potentially harmful biomedical research.

The justifications proposed for invasive chimpanzee experimentation rely upon the important contributions advocates claim it has made toward the advancement of biomedical knowledge, and, in particular, toward combating major human diseases. However, a recent large-scale citation analysis of the medical utility of chimpanzee experimentation indicated that the benefits conferred are significantly less than sometimes claimed. Half of the randomly-selected published chimpanzee studies were not cited by any subsequent papers, apparently generating data of questionable value, which made little obvious contribution toward the advancement of biomedical knowledge. Additionally, closer examination failed to identify any chimpanzee study that made an essential contribution, or, in a clear majority of cases, a significant contribution of any kind, toward papers describing methods efficacious in combating human diseases [51].

Almost all of these chimpanzee experiments would have been approved by at least one institutional ethics committee ethically obliged to allow only those experiments likely to result in substantial benefits, given the considerable animal welfare, bioethical and financial costs integral to chimpanzee experimentation. Whilst the concept of ethical review is sound, these results demonstrate that its implementation is presently flawed. This flaw appears to have resulted from an over-reliance on the assumption that these chimpanzee experiments were likely to be of substantial use in advancing biomedical progress. The approval of large numbers of these experiments, despite their questionable value, indicates a widespread failure of the ethics committee system. By approving these experiments on the basis of unfounded assumptions about their likely benefits, the ethics committees responsible failed in their duty to society, and to the animals they were charged with protecting.

Numerous experts have previously called for the banning of great ape experimentation [6,8,95,109,110]. By 2008, legislative or policy bans or restrictions were in place within seven European countries, Japan, Australia and New Zealand. The revision of *Directive 86/609/EEC on the Protection of Animals used for Experimental and Other Scientific Purposes *may result in the extension of such restrictions to include the remainder of Europe.

In continuing to conduct invasive experiments on chimpanzees and other great apes, by 2008 the US was almost completely isolated internationally. However, the 2007 NCRR permanent moratorium on chimpanzee breeding [2,3], and the 2008 congressional introduction of *The Great Ape Protection Act *[1], may signal a noteworthy change in policy, and have provided an unprecedented opportunity for US legislators, researchers and others, to work toward a future global ban on invasive chimpanzee research.

Previous NIH support of invasive chimpanzee research has been dogged by controversy [5], and chimpanzee experimentation consumes enormous resources that are consequently unavailable to other, potentially more beneficial research fields. Ending such research within the US would uphold the best interests of chimpanzees and other fields presently deprived of research funding, and would also increase the compliance of US animal researchers with internationally-accepted animal welfare and bioethical standards. It could even result in the first global moratorium on invasive research, for any non-human species, unless conducted in the best interests of the individual or species.

## About the author

London-based veterinarian Andrew Knight is the Director of Animal Consultants International [111], which provides multi-disciplinary expertise on animal issues. He founded the organization in 2004 to facilitate increased effectiveness of animal advocacy campaigns worldwide, via international skill-sharing. Andrew is interested in a range of bioethical issues, and has published a series of studies critically assessing the human clinical and toxicological utility of animal experimentation, and reviewing the use of non-animal methodologies, within fundamental research, toxicity testing and biomedical education.
